# Individualized diagnosis of major depressive disorder via multivariate pattern analysis of thalamic sMRI features

**DOI:** 10.1186/s12888-021-03414-9

**Published:** 2021-08-20

**Authors:** Hanxiaoran Li, Sutao Song, Donglin Wang, Zhonglin Tan, Zhenzhen Lian, Yan Wang, Xin Zhou, Chenyuan Pan

**Affiliations:** 1 Institutes of Psychological Sciences, College of Education, Hangzhou Normal University, 2318#, Yuhangtang Rd, Hangzhou, 311121 China; 2 Center for Cognition and Brain Disorders, Hangzhou Normal University, Hangzhou, 311121 China; 3 Zhejiang Key Laboratory for Research in Assessment of Cognitive Impairments, Hangzhou, 311121 China; 4 School of Information Science and Engineering, Shandong Normal University, 1#, University Rd, Changqing District, Jinan, 250358 China; 5 Department of Psychiatry, The Affiliated Hospital, Hangzhou Normal University, Hangzhou, 310015 China; 6 Department of Psychiatry, Hangzhou Seventh People’s Hospital, Hangzhou, 310013 China

**Keywords:** Major depressive disorder (MDD), Thalamus, Multivariate pattern analysis (MVPA), Individualized diagnosis

## Abstract

**Background:**

Magnetic resonance imaging (MRI) studies have found thalamic abnormalities in major depressive disorder (MDD). Although there are significant differences in the structure and function of the thalamus between MDD patients and healthy controls (HCs) at the group level, it is not clear whether the structural and functional features of the thalamus are suitable for use as diagnostic prediction aids at the individual level. Here, we were to test the predictive value of gray matter density (GMD), gray matter volume (GMV), amplitude of low-frequency fluctuations (ALFF), and fractional amplitude of low-frequency fluctuations (fALFF) in the thalamus using multivariate pattern analysis (MVPA).

**Methods:**

Seventy-four MDD patients and 44 HC subjects were recruited. The Gaussian process classifier (GPC) was trained to separate MDD patients from HCs, Gaussian process regression (GPR) was trained to predict depression scores, and Multiple Kernel Learning (MKL) was applied to explore the contribution of each subregion of the thalamus.

**Results:**

The primary findings were as follows: [1] The balanced accuracy of the GPC trained with thalamic GMD was 96.59% (*P < 0.001*). The accuracy of the GPC trained with thalamic GMV was 93.18% (*P < 0.001*). The correlation between Hamilton Depression Scale (HAMD) score targets and predictions in the GPR trained with GMD was 0.90 (*P < 0.001, r*^*2*^ *= 0.82*), and in the GPR trained with GMV, the correlation between HAMD score targets and predictions was 0.89 (*P < 0.001, r*^*2*^ *= 0.79*). [2] The models trained with ALFF and fALFF in the thalamus failed to discriminate MDD patients from HC participants. [3] The MKL model showed that the left lateral prefrontal thalamus, the right caudal temporal thalamus, and the right sensory thalamus contribute more to the diagnostic classification.

**Conclusions:**

The results suggested that GMD and GMV, but not functional indicators of the thalamus, have good potential for the individualized diagnosis of MDD. Furthermore, the thalamus shows the heterogeneity in the structural features of thalamic subregions for predicting MDD. To our knowledge, this is the first study to focus on the thalamus for the prediction of MDD using machine learning methods at the individual level.

**Supplementary Information:**

The online version contains supplementary material available at 10.1186/s12888-021-03414-9.

## Background

Major depressive disorder (MDD) is a common disorder that is associated with a series of clinical symptoms, such as depressed mood, loss of energy, difficulty with concentration and short-term memory and decision making, etc. [1]. As a mental disorder, MDD may create an enormous burden and harm for patients and society. Current clinical diagnostic approaches for MDD are based mainly on the subjective assessment of symptoms through clinician interviews with patients. Psychiatric diagnosis depends largely on statements by the patients and their relatives, psychometrists’ use of many rating scales, and psychiatrists’ personal experiences. These diagnostic methods, however, do not involve any biological or physiological markers and therefore are not objective enough, which may lead to misdiagnosis [2]. To avoid misdiagnosis and achieve better treatment outcomes, objective and individualized diagnostic approaches are urgently needed.

In searching for biomarkers useful for objective diagnosis of MDD, many studies have contributed a lot to the identification of biological correlates of MDD patients in recent years [3–5]. From a special perspective, our recent study demonstrated abnormalities in thalamus in MDD patients [6]. Also, results from many other studies suggest that thalamic abnormalities might be important potential biomarkers of MDD [7–9]. The clinical symptoms of MDD may arise, at least in part, through the corresponding dysfunctions of thalamus and thalamus-related neural circuits [10].The thalamus is not only a sensory relay station involved in emotion, memory, and arousal [11], but also plays a central role in the ongoing cortical function [12], and is a key central region, which can integrate all kinds of information being processed by the whole cerebral cortex [13]. Meanwhile, the thalamus is a part of the salience network, which has been proved to have a central role in MDD [14]. Both structural and functional abnormalities of thalamus were found in patients with MDD. Patients with MDD were shown to have reduced fractional anisotropy values in the prefrontal lobe portion of the left anterior thalamic radiation and increased thalamic blood flow velocity compared with healthy people [15–17]. Other studies have demonstrated decreased left thalamic volume, a contracted shape on ventral aspects of the left thalamus and decreased gray matter volume (GMV) in the right thalamus [16, 18, 19] or in the bilateral thalamus [20], and increased gray matter density (GMD) in the thalamus [21] in MDD patients, while some studies have shown larger thalamic volume, which was seen only in first-episode medication-naive patients. The results of these studies on thalamic GMD and GMV are inconsistent, and these results may be affected by age, severity of depression, and treatment. Thalamic structural abnormalities have been found in MDD patients of different ages. In adolescents with MDD, GMV in the thalamus is inversely related to the severity of self-reported symptoms and decreases with age, while healthy adolescents show increases with age [22]. In elderly patients with depression, the volume of the thalamus is smaller than that in normal people [17, 23]. In terms of the severity of depression, although there is no significant correlation between depression scores and brain structure volume, higher depression scores have indicated more thalamic shape abnormalities [17],while some studies demonstrated that the severity of mild depressive symptoms was associated with reduced gray matter volume in the thalamus [24]. Moreover, thalamic abnormalities have been found in people at high risk of depression. In people with subthreshold depression, the GMV in the thalamus was increased [25]. In a study of healthy people with cognitive vulnerability to depression, it was found that these subjects had a smaller right thalamus than MDD patients [19]. In addition, the thalamus may be related to antidepressant therapy. A multiple regression analysis revealed that pretreatment smaller GMV in the left thalamus was associated with a poorer response to electroconvulsive therapy (ECT) and lower fractional amplitude of low-frequency fluctuations (fALFF) in the left thalamus [26]. Moreover, some studies have demonstrated that the thalamus may play an important role in MDD via thalamocortical circuits. A recent review of previous studies reported that thalamocortical circuits are candidates for controlling the activity of the default network, including task-suppression effects [27]. Thalamocortical circuits are anatomically well situated to exert a broad influence within and between cortical networks and to act as modulatory hubs [28]. Additionally, dysregulation of thalamocortical circuits might increase the risk of certain forms of mental illness, including MDD [27]. Using group statistical analysis methods, the abovementioned studies have provided strong evidence that thalamic abnormalities are closely relevant to MDD. Still, it remains unknown whether the thalamus could be used as proper feature to identify MDD patients at individual-level.

To predict individual cases, multivariate pattern analysis (MVPA) techniques could differentiate MDD patients from healthy controls (HCs) using magnetic resonance imaging (MRI) at the individual level. MVPA has been proven to be more sensitive and more informative about the organization of the cortex than univariate analysis with the general linear model (GLM). MVPA provides an investigation of different brain states that may be produced by a cortical field or systems, thus increasing the amount of information decoded from brain activities [29]. In recent years, a considerable number of studies have built support vector machine (SVM) models to predict the diagnosis of MDD or bipolar disorder (BD), MDD onset, refractory MDD patients, and treatment response to different types of antidepressant therapy, including electroconvulsive therapy, medication therapy and cognitive behavioral therapy, with over 70% accuracy by using structural magnetic resonance imaging (sMRI) or resting-state functional magnetic resonance imaging (rs-fMRI) information [30–38]. In addition, Gaussian process classification (GPC) has also been used to recognize MDD, BD, and remitted MDD patients using fMRI (e.g., amplitude of low-frequency fluctuations (ALFF) and fALFF) or sMRI (e.g., GMD) features with over 69% accuracy [7, 39–45]. GPC is a supervised machine learning approach similar to SVM that provides the added benefit of predictive probabilities of class membership [46]. These results illustrate that MVPA methods show outstanding performance in individually discriminating MDD patients from healthy people and patients with other mental disorders.

Although previous MRI studies have demonstrated thalamic abnormalities in MDD and MVPA methods have shown good performance in individually recognizing MDD patients, to date, there has been no research on the individualized diagnosis of MDD using imaging features of the thalamus. Hence the present study is intended to focus on the thalamus and employ MVPA to predict MDD at the individual level. We were to use two MVPA methods, i.e., GPC and Gaussian process regression (GPR), to examine the potential predictive capacity of structural and rs-fMRI features of the thalamus. Besides, to explore which subregions of the thalamus contribute more to the diagnostic classification of MDD, a sparse version of Multiple Kernel Learning (MKL) was to be applied to explore the contribution of each subregion [47]. We hypothesized that the MRI features of the thalamus would be biomarkers for individualized diagnosis of MDD. More specifically, the predictive potential of both GPCs and GPRs trained with two structural features, i.e., GMD and GMV, and two rs-fMRI features, i.e., ALFF and fALFF, of the thalamus would be expected to bring interesting results for this hypothesis. We also hypothesized that the thalamic subregions would contribute differently to the individualized diagnostic classification of MDD.

## Methods

### Participants

In this study, 118 subjects were recruited, including 74 MDD patients (MDD group) and 44 healthy volunteers as a control group (HC group). Previous studies have shown that education level is a strong predictor of MDD and therefore should be strictly controlled for in the data analysis [48–52]. Because it was difficult to match, education level was included as a covariate and controlled for with statistical techniques during data processing in the present study.

MDD patients (49 female and 25 male patients with an average age of 26.53 ± 8.56 years) were recruited from the Department of Psychiatry of the Seventh People’s Hospital of Hangzhou and the Department of Psychiatry of the Second People’s Hospital of Hangzhou. All enrolled patients met the following criteria: [1] met the International Classification of Diseases, 10th Revision (ICD-10) criteria for MDD [2]; had no history of medication or physiotherapy for at least 1 month before recruitment or were taking only selective serotonin reuptake inhibitor (SSRI) antidepressants ≤ 1 week [3]; had a Hamilton Depression Scale (version: 24 Items; HAMD-24) total score ≥ 20; and [4] were 18–65 years of age. There was no restriction on sex.

Healthy subjects (28 female and 16 male subjects with an average age of 29.34 ± 12.42 years) were recruited from universities in Hangzhou and communities near the hospitals by posters and internet announcements. The inclusion criteria were as follows: [1] did not meet the ICD-10 “depression episode” diagnostic criteria, had no family history of mental illness, and had not taken any medications at least 1 month before recruitment [2]; had a HAMD-24 total score ≤ 8; and [3] were aged 18–65 years.

Both MDD and HC subjects were right-handed Han Chinese individuals. Participants were excluded if they met any of the following criteria: a history of or current organic brain diseases, abuse of or dependence on psychoactive substances, schizophrenia or other psychiatric disorders, depressive episodes with psychotic symptoms or suicidal behavior, serious physical diseases, or any contraindications for MRI, and for women, pregnancy or lactation.

This study was approved by the ethics committee of the Institutes of Psychological Sciences, Hangzhou Normal University. All methods were performed in accordance with the relevant guidelines and regulations. All patients’ legally authorized representatives and the controls provided written informed consent before participating in the study procedures.

### MRI data acquisition

Three-dimensional MR imaging was acquired using a GE 3 T scanner (MR750, GE Medical Systems, Milwaukee, WI) with a 32-channel radio frequency coil at the Center for Cognition and Brain Disorders (CCBD), Hangzhou Normal University (HZNU). Foam filling was used to reduce head movement for all subjects. During scanning, the subjects were asked to relax and remain still. Using a magnetization-prepared rapid acquisition gradient-echo sequence, three-dimensional T1-weighted anatomical images were obtained in the sagittal orientation (TR = 9 ms, TE = 3.664 ms, FOV = 240 × 240 mm^2^, matrix = 300 × 300, flip angle = 13°, thickness = 0.8 mm, acquisition time = 13 min 37 s). fMR images were acquired using a gradient-recalled echo-planar imaging sequence (TR = 2000 ms, TE = 22 ms, FOV = 240 × 240 mm^2^, matrix = 96 × 96, flip angle = 77°, slice thickness = 2.5 mm, no interslice gap, and 240 volumes).

### Data processing

#### MRI data preprocessing

All datasets were preprocessed via DPABI_V3.1 (a toolbox for Data Processing & Analysis for Brain Imaging) [53].

Structural data were segmented into GMV, GMD, white matter volume, white matter density, cerebral spinal fluid volume, and cerebral spinal fluid density. “Dartel+segment” was applied for normalization to the Montreal Neurologic Institute (MNI) space. Images were smoothed with an 8-mm full-width at half-maximum (FWHM) Gaussian kernel.

The following procedures were included in the rs-fMRI data preprocessing: [1] removal of first 10 volumes [2]; slice timing correction [3]; head motion correction [4]; coregistration of T1 images to the averaged EPI image [5]; spatial normalization to standard Montreal Neurological Institute (MNI) space using “Dartel+segment” [6]; regression of head motion effects with the Friston-24 parameter model (all the subject’s head motions were lower than our criteria of 2 mm and 2°) and regression of head motion, white matter (WM) and cerebrospinal fluid (CSF); and [7] removal of linear trends.

#### Features used for classification and prediction

DPABI was used to make the whole-thalamus mask [53] and calculate the GMV, GMD, ALFF, and fALFF values. The GMV, GMD, ALFF, and fALFF values in the thalamus were extracted as regression and classification features.

GMV and GMD are the important indicators of brain structure changes. Many MRI studies found that the abnormal brain structure changes in MDD [54, 55]. These two indicators were obtained through segmenting the structural images using “Dartel+segment”.

ALFF and fALFF reflect the neural activity of the brain. The abnormal levels of ALFF and fALFF may be related to MDD [56–59]. ALFF/fALFF, which are important indicators, are used to detect the local intensity of spontaneous fluctuation of the blood-oxygen-level-dependent (BOLD) signal [60], and the change in local intensity of the BOLD signal depends on the spontaneous fluctuation of regional cerebral blood flow. Thus, increases in ALFF/fALFF may indicate excessive neurological activity in the brain, while decreases in ALFF/fALFF may indicate insufficient neurological activity [61–63]. A ratio of the low-frequency amplitude within 0.01–0.1 Hz was computed at each voxel to obtain the ALFF and fALFF. The maps were smoothed by 8-mm FWHM Gaussian kernel.

#### Pattern analysis

**Fig. 1:**
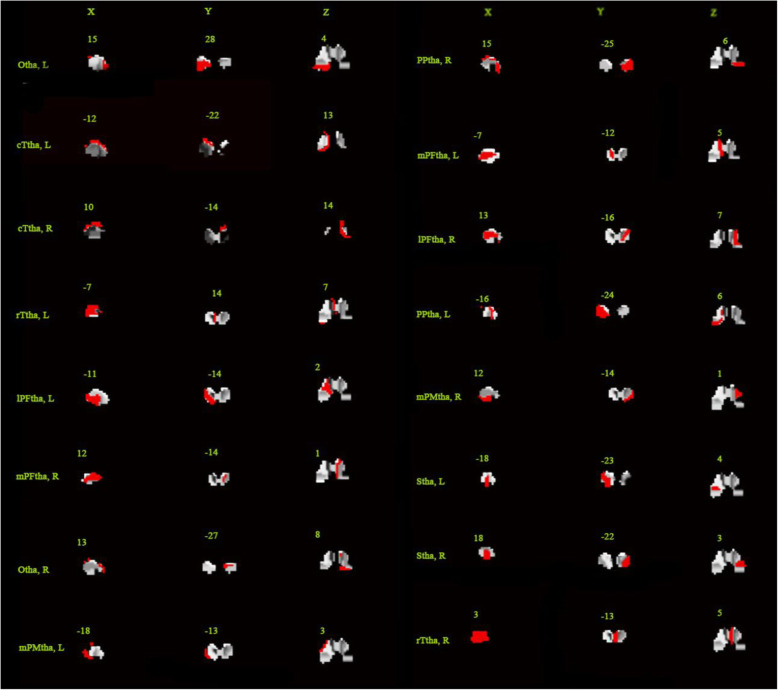
Subregions of the thalamus. mPFtha, medial prefrontal thalamus; mPMtha, premotor thalamus; Stha, sensory thalamus; rTtha, rostral temporal thalamus; PPtha, posterior parietal thalamus; Otha, occipital thalamus; cTtha, caudal temporal thalamus; lPFtha, lateral prefrontal thalamus; L: left; R: right

In this study, GPC was built for pattern classification, and GPR was built for HAMD score prediction using the Pattern Recognition for Neuroimaging data Toolbox (PRoNTo) toolbox (http://www.mlnl.cs.ucl.ac.uk/pronto) [64]. GPR has been widely used in supervised machine learning due to its flexibility and inherent ability to describe uncertainty in function estimation [65]. A mask of the thalamus was firstly added to limit the brain region for analysis, and the BrainnetomeAtlas which divided the thalamus into 16 subregions was added as a secondary mask (see Fig. 1) [66]. For every subregion, the signal in each voxel was extracted and concatenated as a feature vector. A vector was associated to a label (i.e. MDD or HC). Then, a linear kernel was built from the feature vectors for each region. The computed kernels were added to obtain a whole thalamus linear kernel. The kernel and its associated labels were used to train the model and estimate the model parameters. The model can then give an associated predicted label for a new data [47].. No parameters need to be optimized during the model training. Fivefold cross-validation was used to evaluate the generalization performance of the models. Because of the imbalance between the number of MDD patients and HC subjects, balanced accuracy ($ac{c}^{bal}=\frac{1}{C}\sum ac{c}_c$, subscript “c” would be the number of the class) was used to evaluate the performance of each classifier. A 1000-permutation test was performed to determine statistical significance, and cross-validation was repeated for each permutation.

Besides, the MKL model was trained to estimate the contribution of each subregion of the thalamus for the predictive model [47]. The steps of the calculation were similar with the GPC. After building linear kernels for subregions, these kernels and their associated labels were used to train the model. First, model parameters were estimated to define a decision function per kernel. The weight of each decision function was then estimated to provide a final decision function. The contribution of each subregion for the decision function or predictive model can be explicitly computed [47]. We performed the MKL with the soft-margin parameters taking the default values 0.01, 0.1, 1, 10, and 100. The evaluation of the model was the same as GPC and GPR.

## Results

### Sample characteristics

Table 1 shows the demographic variables and clinical characteristics of the two groups. Age (*Z = -0.83, P = .410*) and sex (*χ*^*2*^ *= 0.08, P = .776*) in the MDD group and the HC group were well matched, and there was no significant difference between them according to the Mann-Whitney test and chi-square test, respectively. Because the level of education was significantly higher in the HC group than in the MDD group, which may have potential effects on the results, the level of education was used as an influencing factor for the covariate analysis in all subsequent steps. HAMD-24 scores were also significantly higher in the patient group than in the HC group.

**Table 1:** Demographic and Clinical Characteristics of Subjects

Characteristic	MDD (*n* = 74)	HC (*n* = 44)	Statistic	*P*-Value
Age (Years)	26.53 ± 8.56	29.34 ± 12.42	*Z* = -0.83	.410
Sex, n (%)				
Female	49 (66.22)	28 (63.64)	*χ*^2^ = 0.08	.776
Male	25 (33.78)	16 (36.36)		
Education level	4.68 ± 0.74	5.43 ± 0.73	*χ*^2^ = 39.24	<.001
HAMD-24 score	28.42 ± 6.22	1.36 ± 1.37	t = 36.01	<.001

*MDD* major depressive disorder group, *HC* healthy control group

Education level: 1 (illiterate), 2 (primary school), 3 (junior high school), 4 (senior high school), 5 (college or university), 6 (master’s degree), 7 (doctorate)

*HAMD-24* Hamilton Depression Scale, 24-Item version

### Group-level results

#### Structural differences between MDD participants and HCs

**Fig. 2:**
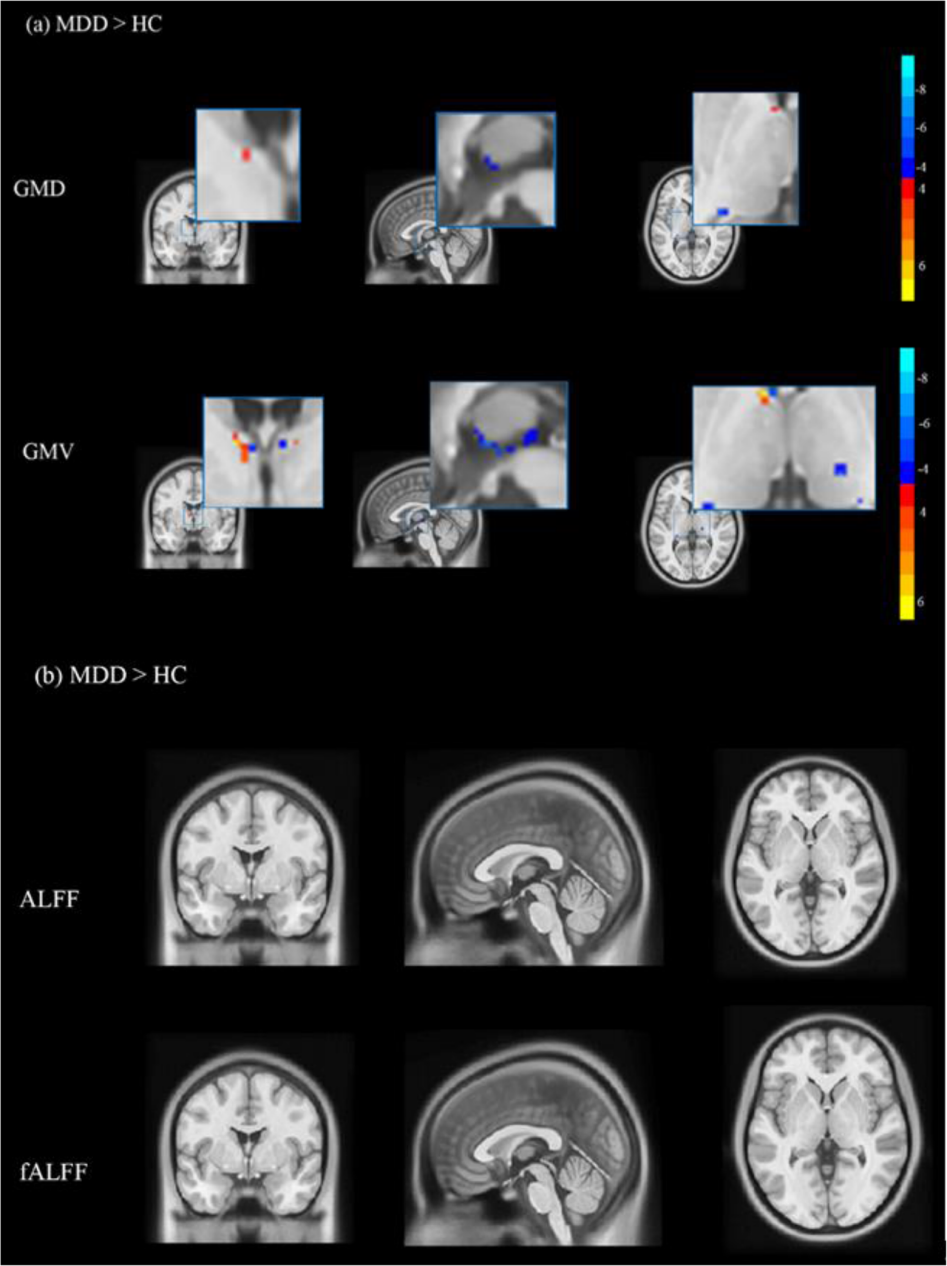
(**a**). The picture shows significant differences between the MDD patients and HC participants in thalamic GMD and GMV. (**b**) The picture shows no significant differences between the MDD patients and HC participants in ALFF and fALFF in the thalamus (Gaussian random field-corrected, voxel *p*-value = 0.001, cluster p-value = 0.05)

Both GMD and GMV in the thalamus in MDD participants were significantly different from those in HC subjects. MDD patients were confirmed to have higher GMD in the left rostral temporal thalamus and lower GMD in the right occipital thalamus and sensory thalamus than HC subjects; MDD patients exhibited higher GMV in the left lateral prefrontal thalamus, the right posterior parietal thalamus, and the right rostral temporal thalamus and lower GMV in the right medial prefrontal thalamus, the right sensory thalamus, and the left rostral temporal thalamus than HCs (see Table 2, Table 3 and Fig. 2a).

**Table 2:** Clusters in the thalamus with abnormal GMD in the MDD patients relative to the healthy controls

Cluster location	Cluster size	MNI			GMD		T value	*P*-Value
		x	y	z	MDD	HC		
rTtha (L)	9	−9	1.5	6	0.55 ± 0.04	0.47 ± 0.04	9.80	< 0.0001
Otha (R)	4	25.5	−33	0	0.38 ± 0.06	0.55 ± 0.08	−12.52	< 0.0001
Stha (R)	2	16.5	−24	3	0.35 ± 0.02	0.41 ± 0.02	−17.85	< 0.0001

*MDD* major depressive disorder group, *HC* healthy control group, *GMD* gray matter density, *rTtha* rostral temporal thalamus, *Otha* occipital thalamus, *Stha* sensory thalamus, *L* left, *R* right

**Table 3:** Clusters in the thalamus with abnormal GMV in the MDD patients relative to the healthy controls

Cluster location	Cluster size	MNI			GMV		T value	*P*-Value
		x	y	z	MDD	HC		
lPFtha (L)	53	−3	−19.5	−4.5	0.29 ± 0.03	0.27 ± 0.02	4.84	<.0001
PPtha (R)	12	9	−31.5	0	0.32 ± 0.05	0.24 ± 0.04	9.99	<.0001
mPFtha (R)	12	6	−4.5	0	0.19 ± 0.03	0.23 ± 0.04	−6.55	<.0001
Stha (R)	31	16.5	−24	4.5	0.27 ± 0.02	0.33 ± 0.03	−12.24	<.0001
rTtha (L)	58	−4.5	1.5	3	0.24 ± 0.04	0.22 ± 0.03	2.75	.007

*MDD* major depressive disorder group, *HC* healthy control group, *GMV* gray matter volume, *lPFtha* lateral prefrontal thalamus, *PPtha* posterior parietal thalamus, *mPFtha* medial prefrontal thalamus, *Stha* sensory thalamus, *rTtha* rostral temporal thalamus, *L* left, *R* right

#### Rs-fMRI differences between MDD patients and HCs

No clusters verified significant differences between MDD patients and HCs in ALFF or fALFF in the thalamus, as shown in Fig. 2b.

### Individual-level prediction: MDD vs. HC participants

#### GPCs trained with sMRI features

**Fig. 3:**
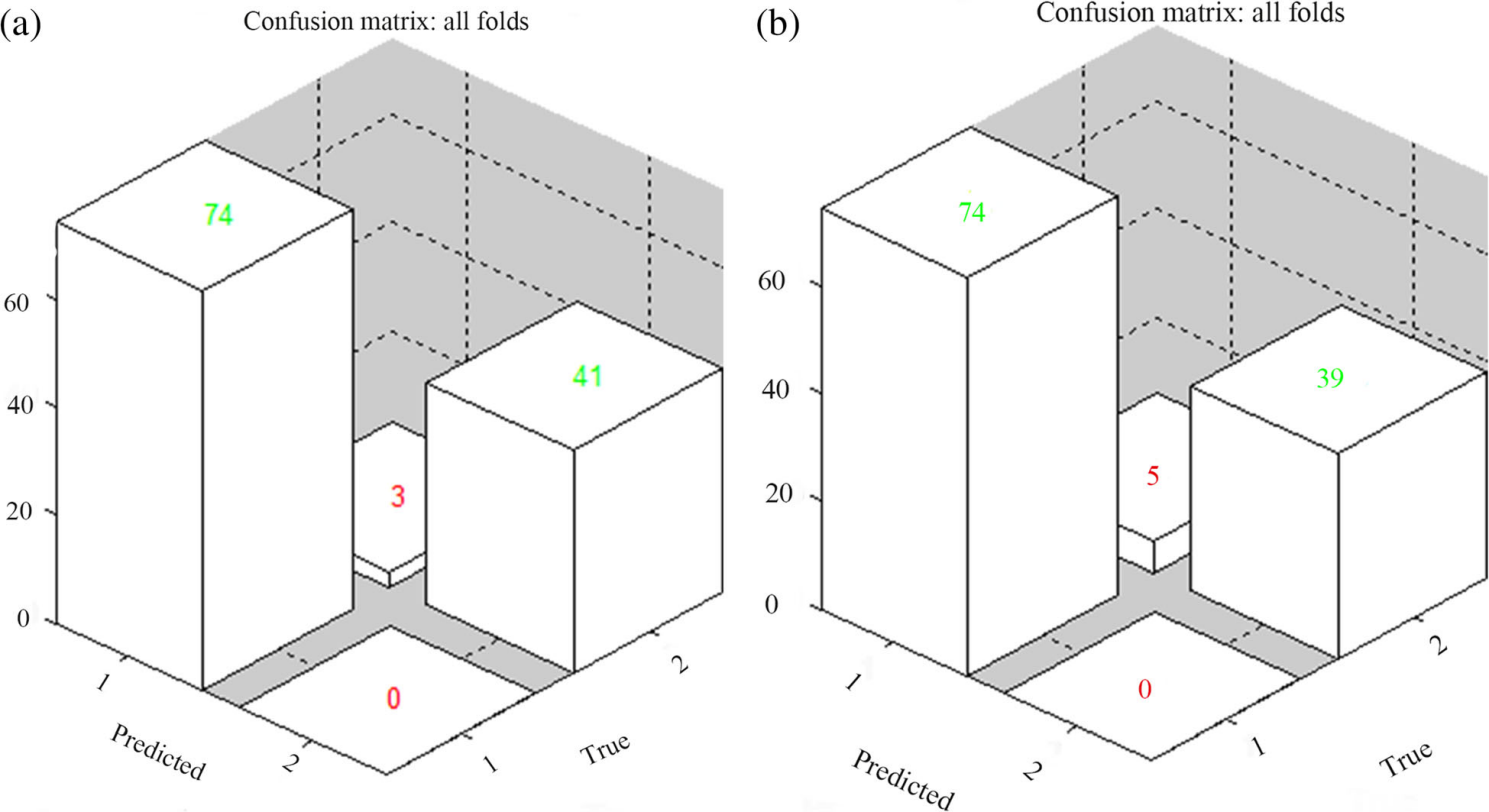
The left image (**a**) shows the classification performance using gray matter density (GMD) data in the thalamus: the balanced accuracy was 96.59%, the sensitivity was 100%, and the specificity was 93.18%. The right image (**b**) shows the classification performance using gray matter volume (GMV) data in the thalamus: the balanced accuracy was 93.18%, the sensitivity was 100%, and the specificity was 86.36%

The GMD and GMV of the thalamus were used to train the GPC. The accuracy of GPC based on GMD of the thalamus was 96.59% (*P < .001*), the sensitivity was 100%, and the specificity was 93.18%. The accuracy of GPC trained with thalamic GMV was 93.18% (*P < .001*), and the sensitivity and specificity were 100% and 86.36%, respectively (see Table 4 and Fig. 3).

**Table 4:** The performance of GPCs trained with GMD and GMV

Indicators	Balanced accuracy (%)	BA *p*-value	Sensitivity (%)	Specificity (%)
GMD	96.59	<0.001	100.00	93.18
GMV	93.18	<0.001	100.00	86.36

*GPC* Gaussian process classification, *GMD* gray matter density, *GMV* gray matter volume

#### MKLs trained with sMRI features

The accuracy of MKL based on GMD of the thalamus was 97.73% (*P < .001*), the sensitivity was 100%, and the specificity was 95.45%. The accuracy of MKL trained with thalamic GMV was 98.86% (*P < .001*), and the sensitivity and specificity were 100 and 97.73%, respectively (see Table 5). The contribution of each subregion to the classification is shown in Table 6.

**Table 5:** The performance of MKLs trained with GMD and GMV

Indicators	Balanced accuracy (%)	BA *p*-value	Sensitivity (%)	Specificity (%)
GMD	97.73	<0.001	100.00	95.45
GMV	98.86	<0.001	100.00	97.73

*MKL* Multiple Kernel Learning, *GMD* gray matter density, *GMV* gray matter volume

**Table 6:** The weights of thalamic subregions for MKLs

GMD		GMV	
Location	Weights (%)	Location	Weights (%)
lPFtha, L	32.87	lPFtha, L	35.31
cTtha, R	23.53	cTtha, R	28.84
Stha, R	12.97	Stha, R	13.29
mPMtha, R	11.28	rTtha, L	12.56
rTtha, L	9.97	mPMtha, L	4.29
mPMtha, L	7.21	Otha, R	3.40
rTtha, R	2.18	mPMtha, R	1.81
mPFtha, L	0.00	Stha, L	0.26
mPFtha, R	0.00	Otha, L	0.24
Stha, L	0.00	mPFtha, L	0.00
PPtha, L	0.00	mPFtha, R	0.00
PPtha, R	0.00	rTtha, R	0.00
Otha, L	0.00	PPtha, L	0.00
Otha, R	0.00	PPtha, R	0.00
cTtha, L	0.00	cTtha, L	0.00
lPFtha, R	0.00	lPFtha, R	0.00

*MKL* Multiple Kernel Learning, *GMD* gray matter density, *GMV* gray matter volume, *mPFtha* medial prefrontal thalamus, *mPMtha* premotor thalamus, *Stha* sensory thalamus, *rTtha* rostral temporal thalamus, *PPtha* posterior parietal thalamus, *Otha* occipital thalamus, *cTtha* caudal temporal thalamus, *lPFtha* lateral prefrontal thalamus, *L* left, *R* right

#### GPCs trained with rs-fMRI features

**Fig. 4:**
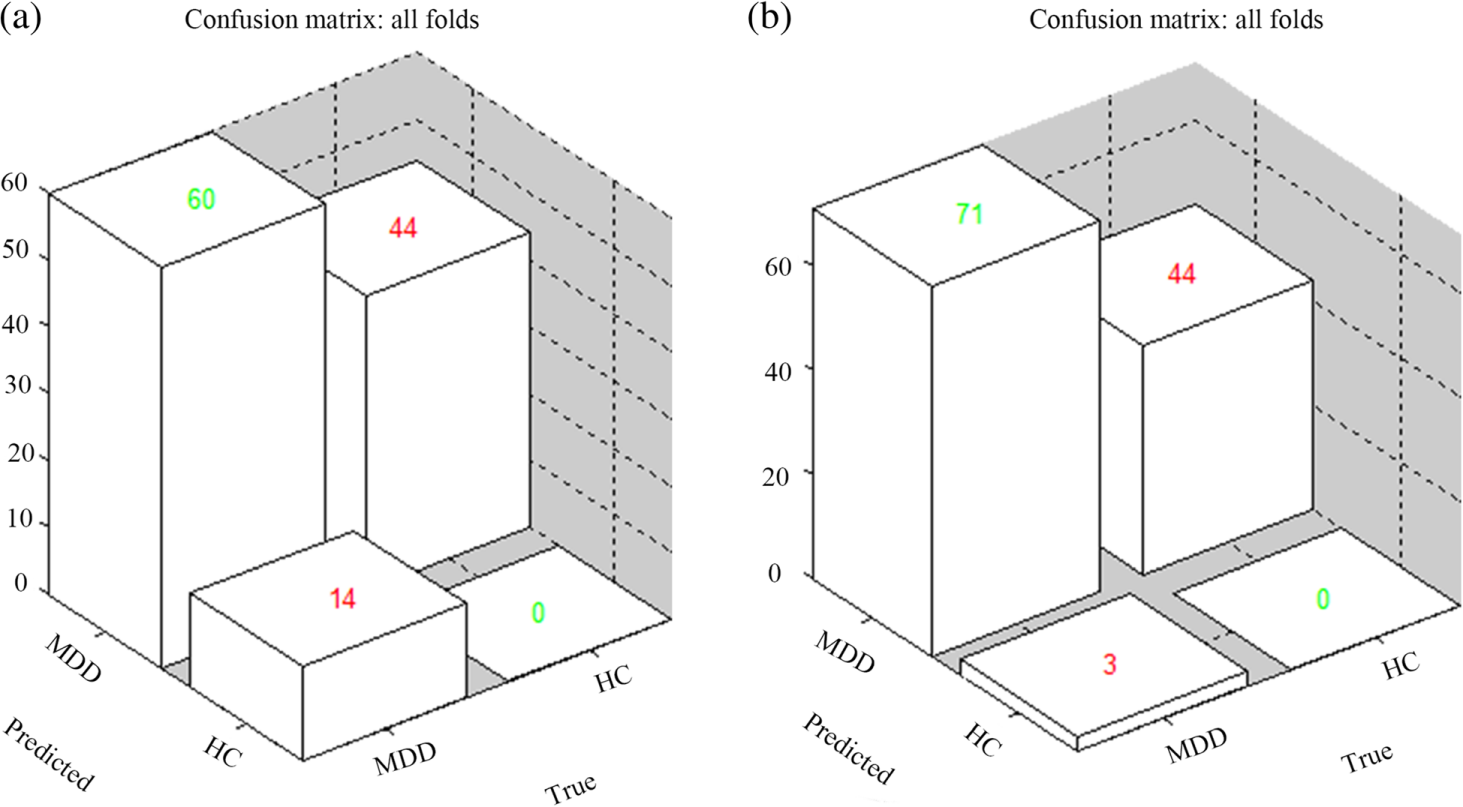
The left image (**a**) shows the classification performance using amplitude of low-frequency fluctuation (ALFF) data in the thalamus: the balanced accuracy was 40.54%, the sensitivity was 81.08%, and the specificity was 0.00%. The right image (**b**) shows the classification performance using fractional amplitude of low-frequency fluctuation (fALFF) data in the thalamus: the balanced accuracy was 47.97%, the sensitivity was 95.95%, and the specificity was 0.00%

The accuracy of GPCs trained with ALFF and fALFF was at the chance level (see Table 7 and Fig. 4). The accuracy of GPC trained with ALFF in the thalamus was 40.54% (*P = .808*), and the accuracy of GPC trained with fALFF in the thalamus was 47.97% (*P = .534*).

**Table 7:** The performance of GPCs trained with ALFF and fALFF

Indicators	Balanced accuracy (%)	BA *p*-value	Sensitivity (%)	Specificity (%)
ALFF	40.54	0.808	81.08	0.00
fALFF	47.97	0.534	95.95	0.00

*GPC* Gaussian process classification, *ALFF* amplitude of low-frequency fluctuations, *fALFF* fractional amplitude of low-frequency fluctuations

### Individual-level prediction of HAMD scores

#### GPRs trained with sMRI features

**Fig. 5:**
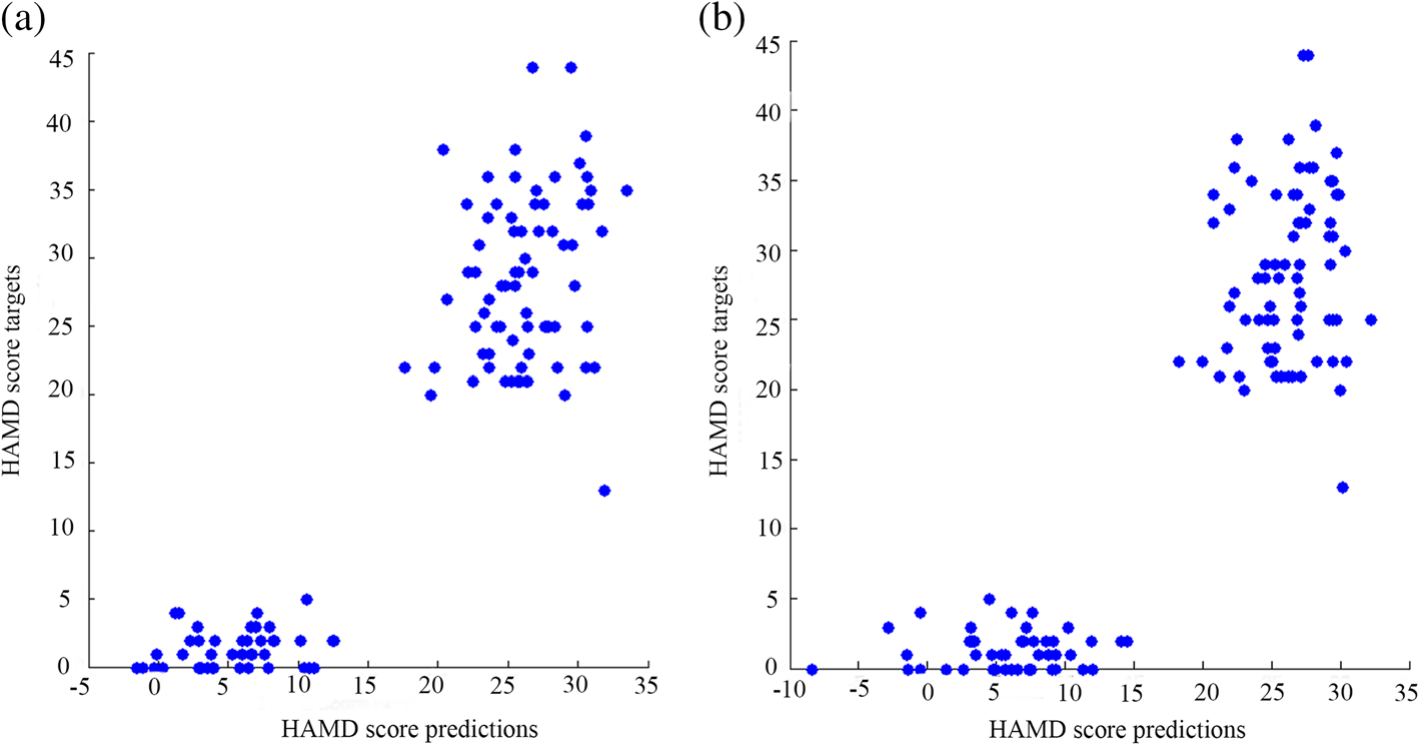
HAMD score targets and HAMD score predictions. “HAMD score targets” means “the actual HAMD score”. The left graph (**a**) shows the performance of the GPR trained with the GMD in the thalamus, and the right graph (**b**) shows the performance of the GPR trained with the GMV in the thalamus

This study built a GPR model that used gray matter information to predict the HAMD scores of participants. The correlation between HAMD score targets and predictions in the GPR trained with the GMD of the thalamus was 0.90, the *P*-value was lower than 0.001, and the coefficient of determination r^2^ = 0.82. In a GPR model trained with the GMV of the thalamus, the correlation between HAMD score targets and predictions was 0.89, the *P*-value was lower than 0.001, and r^2^ = 0.79. Figure 5 shows the results.

#### GPRs trained with rs-fMRI features

**Fig. 6:**
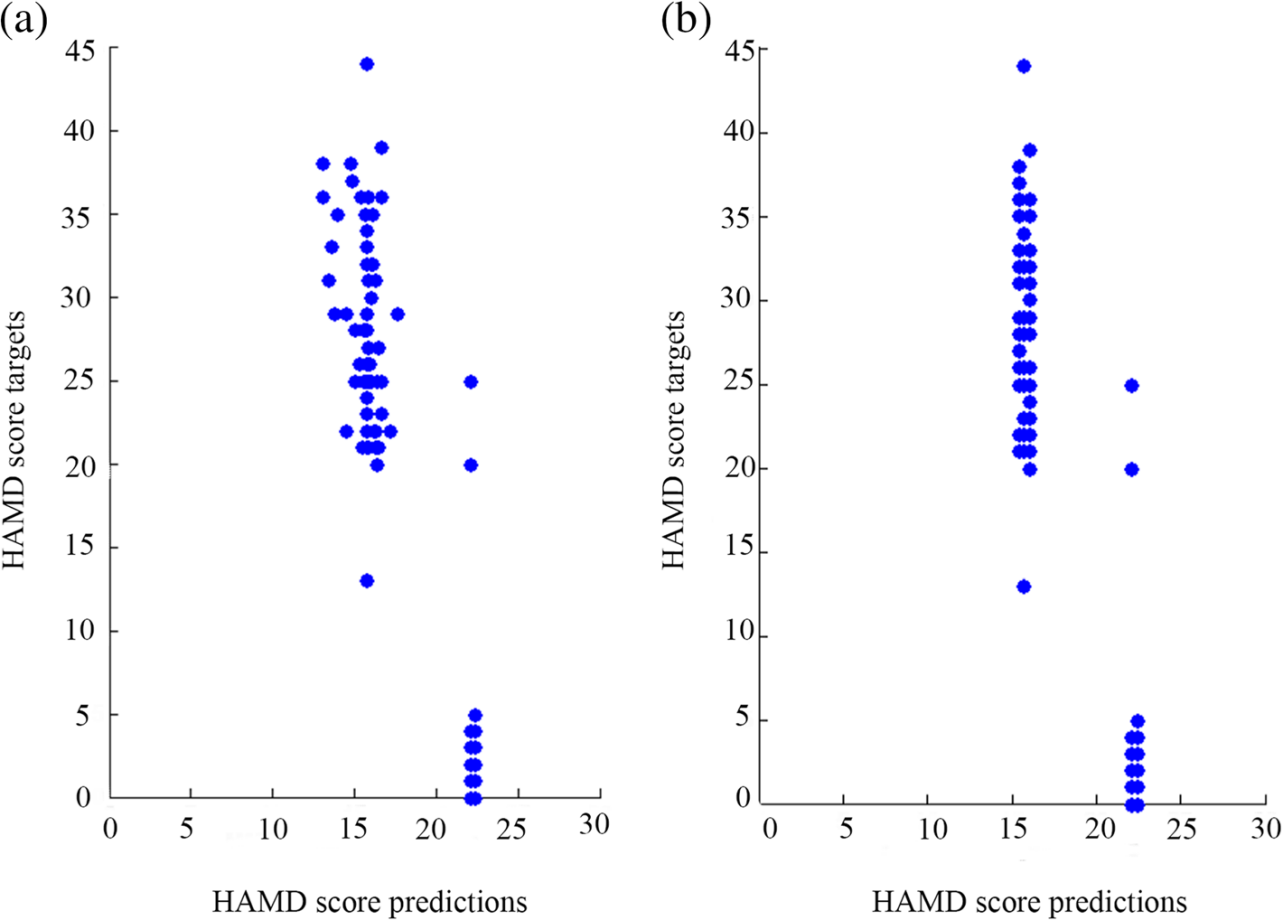
The left graph (**a**) shows the performance of the GPR trained with ALFF in the thalamus, and the right graph (**b**) shows the performance of the GPR trained with fALFF in the thalamus

The GPR models trained with rs-fMRI data showed a negative correlation between the true HAMD scores and predictions, which implied that the GPR models cannot correctly predict the HAMD scores. In the GPR trained with ALFF in the thalamus, the correlation between HAMD score targets and predictions was − 0.92 (*P = .640, r*^*2*^ *= 0.84*). The correlation between targets and predictions in the GPR trained with fALFF of the thalamus was − 0.92 (*P = .872, r*^*2*^ *= 0.84*). The results are shown in Fig. 6.

## Discussion

In this study, we investigated the potential capacity of the two structural features (i.e., GMD and GMV) and the two rs-fMRI features (i.e., ALFF and fALFF) of the thalamus in the diagnosis of MDD at the individual level by MVPA methods (i.e., GPC and GPR). The results showed that the balanced accuracy of the machine learning models trained with thalamic GMD and GMV was significantly higher than the chance level. The correlation between the real and the predicted HAMD scores in the GPRs trained with GMD and GMV was significant. The results also showed that the models trained with ALFF and fALFF in the thalamus failed to discriminate MDD patients from HC participants. Findings from this study suggest that the structural MRI features rather than the rs-fMRI features of the thalamus may have good potentials for the individualized diagnosis of MDD.

This study confirms that the thalamus is closely related to MDD, and different machine learning models (i.e., GPC and GPR) trained with thalamic gray matter imaging indicators showed good performance in identifying MDD patients, which corresponded with our group-level results showing significantly different clusters in the thalamus. It is well known that all sensory nerve pathways, except for those conveying olfactory information, project to the thalamus [67]. In other words, the thalamus is a sensory relay station that is involved in emotion, memory, and arousal [11]. Some evidence has been illustrated to support the argument that the thalamus is not simply a relay station [13] but also plays a central role in ongoing cortical functioning [12]. The thalamus is globally connected with distributed cortical regions, most thalamic subdivisions display network properties that are capable of integrating multimodal information across diverse cortical functional networks, and the thalamus is involved in multiple cognitive functions [13]. Additionally, evidence has suggested that the human thalamus is a critical hub region that could integrate diverse information being processed throughout the cerebral cortex [13]. The thalamus relays this information to the corresponding cerebral cortical areas and from there to the amygdala and hippocampus, which are the regions of the brain most closely related to emotion, memory, and arousal [11]. Depressed mood, loss of energy, difficulty with short-term memory, etc. are included in the core symptoms of a depressive episode [68]. Furthermore, a recent review of previous studies confirmed that the results of rodent studies indicate that thalamocortical circuits are candidates for controlling the activity of the default network, including task-suppression effects [27]. Dysregulation of thalamocortical circuits might also increase the risk of certain forms of mental illness [27]. MRI studies have demonstrated that MDD patients have abnormalities in prefrontal, temporal, parietal, insular, occipital, and subcortical structures [54, 55]. The abovementioned brain areas are all related to thalamocortical circuits. If the gray matter in the thalamus, an important part of thalamocortical circuits, is abnormal, it may cause the whole thalamocortical circuit to be abnormal, which may lead to MDD [27]. Therefore, the analysis of structural imaging data from the thalamus could distinguish MDD patients from healthy people. Such an analysis, when performed at the individual level by employing MVPA as in our study, is more valuable for predicting individual cases.

The group-level analysis of the gray matter features of the thalamus reported significantly different clusters between the MDD patients and HCs. Concurrently, the subregions where most of the clusters are located had high contribution weights in the classification. For the first time, we found that heterogeneity in the thalamus at the subregional level identified individuals with depression. The thalamus comprises numerous nuclei, which project to different brain areas and receive inputs from other cortical or subcortical brain regions [11, 13]. The difference in connections between the different thalamic subregions and other brain regions may be associated with different functions in thalamic subregions. The medial dorsal nucleus of the thalamus may play a role in memory (perhaps specifically in the retrieval of episodic memory), mood, motivation, and the sleep/wake cycle [11]. The anterior nucleus of the thalamus may be involved in memory, modulation of the sleep/wake cycle, and directed attention [11]. The lateral dorsal nucleus of the thalamus may be related to motivation and/or attention with sensory processes [11]. Thus, this heterogeneity may explain the differences in the results across thalamic subregions. The findings of heterogeneity across thalamic subregions were indirectly supported by the results of our previous study [6], which, through functional imaging data, revealed that MDD patients exhibited distinct resting-state functional connectivity patterns across thalamic subregions.

Results from this study suggest that ALFF and fALFF in the thalamus may not be robust features for recognizing MDD patients. No significant difference in ALFF or fALFF clusters in the thalamus was found between the MDD and HC groups, and machine learning models trained with ALFF and fALFF in the thalamus failed to effectively discriminate individual patients from healthy people with ideal performance in this study. We also performed a two-sample t-test on the whole brain and found that the differences of ALFF and fALFF were located in the cortex area and brainstem, not the thalamus (see supplementary materials Fig. S1). ALFF, in which the square root of the power spectrum was integrated in a low-frequency range, was used to detect the regional intensity of spontaneous fluctuations in the BOLD signal [69]. In fALFF, the ratio of the power spectrum of the low-frequency (0.01–0.08 Hz) range to that of the entire frequency range was computed [60]. Most neuroimaging MDD studies have reported abnormal ALFF and fALFF levels in the left cerebellum, amygdala, left hippocampus, precuneus, right cingulate cortex, right putamen, medial prefrontal cortex, left motor cortex and parietal lobe [56–59], and some researches have found that abnormal thalamic ALFF or fALFF may correlate with the antidepressant response but not MDD onset [9]. Another reason why this study showed negative ALFF and fALFF results in the thalamus was that a few days before enrollment some of the MDD patients were taking antidepressants, which may have influenced the research results. Some studies have reported that ALFF and fALFF could be changed by antidepressant use [70]. Thus, the reason why the ALFF and fALFF features of the thalamus did not have good enough performance in discriminating MDD individuals in our study may in part be related to this.

To our knowledge, this is the first study to focus on the thalamus for the individualized diagnosis of MDD. Using machine learning methods to analyze the MRI data of thalamus, this study established an individualized brain morphology-related diagnostic model for MDD based on thalamic imaging features. If this model could be applied in clinic, it is expected to be helpful to improve the current situation that the diagnosis of MDD in psychiatric clinic depends mainly on patients’ self-statement and psychiatrists’ subjective judgment, and also helpful to reduce the risk of misdiagnosis of MDD. Further, the results of this study not only may provide an important basis for the early identification and objective diagnosis of MDD at individual level, but also may provide useful clues for the exploration of the biological and pathological mechanism behind MDD. Additionally, our study reveals for the first time the heterogeneity in the structural features of thalamic subregions for predicting MDD at the individual level, which demonstrates the importance and the heterogeneity of the thalamus in MDD, and may provide some clue for further research about the whole thalamus and thalamic subregions in emotion-related disorders.

There are some limitations in this study: [1] Although 118 subjects were included in this study, which exceeded the sample size of most previous single-center studies, the sample size of this study was still not large enough (from the perspective of requirements of multivariate pattern analysis method), which may lead to some deviation between the classification results and the actual situation. We know that small data sets may possibly lead to overfitting. By building a larger database upon which to base a predictive model, the variations observed among MDD patients could be more thoroughly incorporated, which, in the future, may result in models with better clinical utility [71]. In future studies, it will be necessary to explore the validation of the performance of GPC and GPR in an independent large database [2]. Not all MDD patients in our study were medication-free subjects, and some of them were not in their first depressive episode. This may have had some influence on the results of this study. These problems need to be addressed in future studies [3]. In this study, the machine learning models trained with ALFF and fALFF in the thalamus failed to effectively discriminate individual MDD patients from healthy persons. This definitely does not mean that there are no functional alterations in the thalamus in MDD. Nor does it mean that other functional MRI data of the thalamus are not suitable to be chosen as features for individualized recognition of MDD patients. One of our previous studies showed sample entropy changes in the bilateral thalami in MDD patients [6], so we are considering using the sample entropy of the resting-state fMRI data as a feature for the classification and prediction of MDD in our future studies [4]. The whole brain volume was not controlled as a covariate in this study, which may have potential correlation with the volume of the thalamus, and could be considered in future studies.

## Conclusions

In conclusion, this is the first study to focus on the thalamus and to use machine learning methods to differentiate MDD patients from healthy people. Both classifiers trained with gray matter volume data and gray matter density data have been confirmed to have high discriminatory accuracy by pattern analysis. Both GPRs trained with the GMD and the GMV in the thalamus could predict HAMD scores of the participants. The GPCs and GPRs trained with ALFF and fALFF in the thalamus showed poor performance in recognizing MDD patients. Therefore, the results of this study suggest that gray matter information, but not functional information, in the thalamus has good potential for the individualized diagnosis of MDD. It would be expected that our results would not only provide important basis for the early identification and objective diagnosis of MDD, but also provide useful clues for the exploration of the biological and pathological mechanism behind MDD.

## Supplementary Information

**Additional file 1.**

## Data Availability

The datasets used and/or analyzed during the current study are available from the corresponding author on reasonable request.

## Abbreviations

MRI: Magnetic resonance imaging
MDD: Major depressive disorder
HCs: Healthy controls
GMD: Gray matter density
GMV: Gray matter volume
ALFF: Amplitude of low-frequency fluctuations
fALFF: Fractional amplitude of low-frequency fluctuations
GPR: Gaussian process regression
GPC: Gaussian process classifier
MVPA: Multivariate pattern analysis
GLM: General linear model
SVM: Support vector machine
BD: Bipolar disorder
sMRI: Structural magnetic resonance imaging
rs-fMRI: Resting-state functional magnetic resonance imaging
CCBD: Center for Cognition and Brain Disorders
HZNU: Hangzhou Normal University
MNI: Montreal Neurological Institute
WM: White matter
CSF: Cerebrospinal fluid
mPFtha: Medial prefrontal thalamus
mPMtha: Premotor thalamus
Stha: Sensory thalamus
rTtha: Rostral temporal thalamus
PPtha: Posterior parietal thalamus
Otha: Occipital thalamus
cTtha: Caudal temporal thalamus
lPFtha: Lateral prefrontal thalamus
L: Left
R: Right
