# Supplementary material for: Individualized diagnosis of major depressive disorder via multivariate pattern analysis of thalamic sMRI features
Source: BMC Psychiatry. 2021 Aug 20;21:415. doi: 10.1186/s12888-021-03414-9 (PMC8377985; doi:10.1186/s12888-021-03414-9)
Supplement: Supplementary file 1 — Additional file 1. [file 12888_2021_3414_MOESM1_ESM.docx]

*
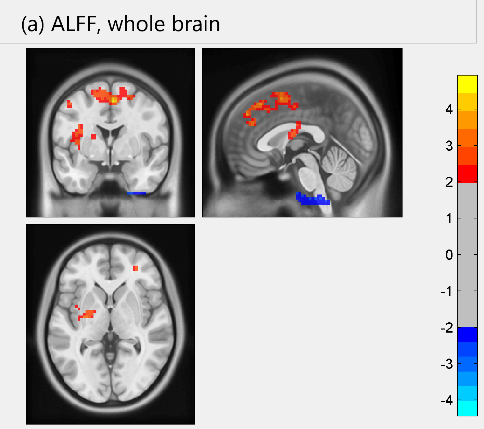

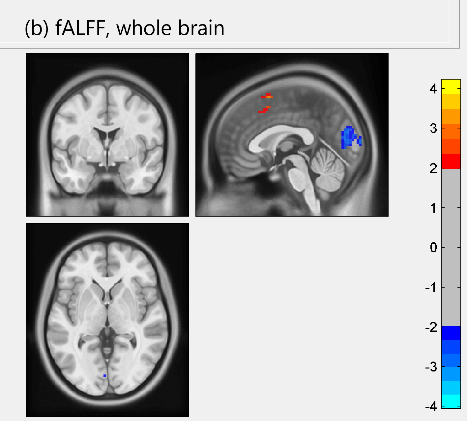
*

Fig. s1 The differences between MDD patients and HC on the whole brain (MDD > HC). We make a two-sample t-test on the whole brain and found that the differences situated in the cortex area and brainstem, not the thalamus
